# Lack of association between *PAX6*/*SOSTDC1*/*FAM20B* gene polymorphisms and mesiodens

**DOI:** 10.1186/s12903-019-0788-3

**Published:** 2019-05-27

**Authors:** Shanshan Liu, Jiancheng Li, Jincheng Xu, Shengkai Liao, Yongfeng Chen, Rongxiu Zhang, Ruixue Tian, Kai Zhang

**Affiliations:** Department of Stomatology, The First Affiliated Hospital of Bengbu Medical College, 287 Chang Huai Road, Bengbu, 233004 China

**Keywords:** Mesiodens, *PAX6*, *SOSTDC1*, *FAM20B*, Genetic polymorphism

## Abstract

**Background:**

The purpose of this study was to analyze the association between the genetic polymorphism of genes (*PAX6*, *SOSTDC1*and *FAM20B*) and the susceptibility to mesiodens.

**Methods:**

This study was carried out on 50 patients with mesiodens and 50 controls. The family history of each patient with mesiodens were recorded. Genomic DNA was extracted from saliva samples, and single nucleotide polymorphisms were detected in all exons and exon/intron boundaries of *PAX6*, *SOSTDC1* and *FAM20B* using Sanger sequencing. The data were analyzed using pearson chi-square test with theoretical frequency ≥ 5. For theoretical frequency less than 5 but at least 1 (≤20% cell), the data were analyzed by continuity correction. For the rest, Fisher’s Exact test was used. A *P*-value< 0.05 was considered statistically significant. The Odds ratio (OR) and confidence intervals (CI) were recorded.

**Results:**

Three polymorphisms were detected in *PAX6*. Two polymorphisms were detected in *SOSTDC1*. Twenty-nine polymorphisms were detected in *FAM20B*. Although, the T allele of *FAM20B* (rs3766626) appears to be associated with mesiodens (*P* = 0.051), there were no significant differences of *PAX6*/*SOSTDC1*/*FAM20B* gene polymorphisms between the two groups. The T allele of *FAM20B* (rs3766626) was associated with susceptibility to two mesiodens (*P* < 0.001; OR = 8.333; CI = 2.516–27.600).

**Conclusions:**

Lack of association between *PAX6*/*SOSTDC1*/*FAM20B* gene polymorphisms and mesiodens in the population studied was detected. Further studies with large samples on T allele of *FAM20B* (rs3766626) are needed.

**Electronic supplementary material:**

The online version of this article (10.1186/s12903-019-0788-3) contains supplementary material, which is available to authorized users.

## Background

Mesiodens is the most common supernumerary teeth located in the central position of the upper or lower jaw [1]. Mesiodens can be either erupted or impacted in alveolar bone placed and oriented vertically, horizontally or in an inverted manner [2, 3]. The prevalence of mesiodens in the population ranges from 0.09 to 2.2%, according to previous studies [4–6]. A series of clinical complications can be caused by mesiodens, including malposition or delayed eruption of the permanent incisors or the formation of a dentigerous cyst [7–9]. However, the etiology of mesiodens is still unclear.

An increasing number of studies indicate that genetic polymorphisms are associated with oral diseases. For instance, in Polish children, the prevalence of the AG genotype of the Enamelin (*ENAM*) gene (rs12640848) was higher in subjects with dental caries compared to that in controls [10]. The polymorphism of COX2 -765G/C had significant influence on periodontitis risk [11]. The genetic polymorphism of axin 2 (*AXIN2*) and Gremiln-2 (*GREM2*, also called *PRDC*) were related with tooth agenesis [12, 13].

Studies have shown that several genes can result in the formation of mesiodens. The Paired box gene 6 (*PAX6*) mutant in rats can result in the formation of a supernumerary upper incisor [14]. The inactivation of Family with sequence similarity member 20-B (*FAM20B*) in the dental epithelium in mice results in supernumerary maxillary and mandibular incisors [15]. The deletion of Sclerostin domain-containing 1 (*SOSTDC 1*, also known as *Wise*, Ectodin, or *USAG-1*) in mice leads to the development of extra molar and incisors [16, 17]. However, research regarding the association between genetic polymorphism and mesiodens formation has been reported less often. Therefore, the purpose of the current study is to analyze the association between mesiodens formation and the genetic polymorphisms of genes related to this process, identifying the importance of genetic polymorphisms in mesiodens formation-related genes.

## Methods

### Study participants

One hundred patients (50 mesiodens group, 50 unrelated controls) were recruited in this study in Bengbu, China. The diagnosis of mesiodens was based on oral examination combined with periapical radiograph, panoramic radiograph, and/ or cone-beam computed tomography. The characteristics including gender, crown direction, the number of mesiodens, and the eruption status of mesiodens were recorded. All patients had no abnormalities in their head, ears, eyes, nose, throat, thyroid, trunk, or extremities and were without cleft lip or palate, congenital absence of teeth or tooth malformation. The family history was recorded.

### Saliva collection and genomic DNA extraction

A total of 2 mL of unstimulated saliva sample for each recruited participant was collected and stored using Oragene DNA Self-Collection kits (Lang Fu, Shanghai, China). Genomic DNA was extracted using the MagBeads Saliva & Swab DNA Extraction Kit (Regular & Pre-loading Version, Enriching Biotechnology LTD, Shanghai, China) according to the manufacturer’s protocol. The Genomic DNA samples were stored at − 20 °C until further analysis.

### Sanger sequencing of selected mesiodens formation related genes

We were particularly interested in *PAX6*, *SOSTDC1*, and *FAM20B*, which were reported to result in the formation of mesiodens. All exons and exon/intron boundaries of these three genes in 100 samples were amplified using a GC-rich PCR Kit (Sangon Biotech, Shanghai, China) combined with Champagne Taq DNA Polymerase (Vazyme, Nanjing, China). The PCR products were purified using a MagBeads Gel DNA Extraction Kit (Enriching Biotechnology LTD, Shanghai, China) according to the manufacturer’s instructions. The PCR reaction mixture (50 μL) included 3 μL of template, 5 μL of buffer, 4 μL of dNTP, 1 μL of each of the specific forward and reverse primers for these three genes, 0.25 μL rTaq enzyme, and RNase-free water. The PCR was performed with the following temperature procedures: denaturation at 94 °C for 5 min, 35 cycles of 30 s at 94 °C, 30 s at 55 °C, and 30 s at 72 °C, with a 10-min extension step at 72 °C. The purified products were used for Sanger sequenced using the ABI Prism 3730 platform (Applied Biosystems™, USA). The primers used for amplification and sequencing are listed in Table 1. The primers of *PAX6* were selected according to previous study [18]. The amplification sequences were detailed in Additional file 1 and Additional file 2.

**Table 1:** Amplification and sequencing primers

ID	Primer sequences	Amplicon size (bp)	Sequencing size (bp)
*PAX6* exon-1 F	CAAACGGACCAATTGCACCA	432	432
*PAX6* exon-1 R	GGTTGGTGTGTGAGAGCAATTCTC		
*PAX6* exon-2 F	CAGAGGTCAGGCTTCGCTAA	449	449
*PAX6* exon-2 R	TCGCTGGAAGTAGAAAGTTTGG		
*PAX6* exon-3 F	TGACTGAGCCCTAGATGCATGTG	466	466
*PAX6* exon-3 R	TCCCCAATCTGTTTCCCCTACAT		
*PAX6* exon-4 F	GAACGGAGATTCTCCTGTCCTA	364	364
*PAX6* exon-4 R	CAGTATCGAGAAGAGCCAAGC		
*PAX6* exon-5 F	AGGATGCATTGTGGTTGTCTCCTC	405	405
*PAX6* exon-5 R	TGGGGGGGTCCATAATTAGCA		
*PAX6* exon-6 F	TCCAAGTGCTGGACAATCAA	707	707
*PAX6* exon-6 R	AGAGGACACAGACTAAGAGACA		
*PAX6* exon-7 F	GGTGTATCTGCAAATCCACCCA	470	470
*PAX6* exon-7 R	CAATGTGGTCGATGTGTCCCA		
*PAX6* exon-8 F	AAGGCTGACAGTTACCTTGGGAA	398	398
*PAX6* exon-8 R	TCTTCTATGCAAAGGGCCCTG		
*PAX6* exon-9 F	TTGGTTGGAGGTAATGGGAGTG	334	334
*PAX6* exon-9 R	TGGCAGCAGAGCATTTAGCAG		
*PAX6* exon 10–11 F	CCTAGAGACAGAGGTGCTTGTA	614	614
*PAX6* exon 10–11 R	GCAGACACAGCCAATGAGG		
*PAX6* exon-12 F	AGCTCGAGGCCCAATCTTAGAT	436	436
*PAX6* exon-12 R	AGGGACAAGGAAAGCAAGGAGT		
*PAX6* exon-13 F1	CTTTTCCTTTGGATTGGGGTG	654	–
*PAX6* exon-13 R1	CACAGATCAAACATCCATCCAGTC		
*PAX6* exon-13 F2	CCTATAAATTTGTATTCCATGTC	Only used for sequencing	149
*PAX6* exon-13 R2	CTTGGCCAGTATTGAGACATATC		
*SOSTDC1* exon-1 F	ACAAGTGATGAAGTCCAACTCT	550	550
*SOSTDC1* exon-1 R	TGTGAGCTAATGCTACCAGAA		
*SOSTDC1* exon-2 F1	TGAAAGTGTCCCTATACTATCC	842	842
*SOSTDC1* exon-2 R1	AACTACAGGATACGTGGAAT		
*SOSTDC1* exon-2 F2	AAATTCCACGTATCCTGTAG	800	800
*SOSTDC1* exon-2 R2	CATGTTAGAGGCAACAACA		
*FAM20B* exon-1 F	GTCCTGCTGCTTGGCTGCCTACCTAC	832	832
*FAM20B* exon-1 R	CCTTCAGCCGCGACCGCACA		
*FAM20B* exon-2 F	ACTGCTGCCATCATTAGGTCC	949	949
*FAM20B* exon-2 R	CAGTGGGTTACCAGGTGTTCT		
*FAM20B* exon-3 F	AATCAGGCTTGCTAATGGGTG	417	417
*FAM20B* exon-3 R	AGGCCAGAAATGAAATGACCTA		
*FAM20B* exon-4 F	TTAATTTGCTCTGTGGGCTTAG	825	825
*FAM20B* exon-4 R	CACCTGCTTTCACCATCACTA		
*FAM20B* exon 5–6 F	AGAGTGAGACTGGGTAGAAAGGA	1130	1130
*FAM20B* exon 5–6 R	TAGCCAAGAAAGAACGATGTAG		
*FAM20B* exon −7 F	AAGTTCTCCCTTTGGTCTGTG	561	561
*FAM20B* exon-7 R	TTTGGGTTATCTGCCTTCAC		
*FAM20B* exon-8 F1	CCATAATTTAACTATTTCCCAGTCG	862	862
*FAM20B* exon-8 R1	CCAATCCCAGTATTCATCTATCC		
*FAM20B* exon-8 F2	TGGTGACGGGACAGAGTGGC	795	795
*FAM20B* exon-8 R2	CAGTTTGCTTTGTTAATTTGGGAAG		
*FAM20B* exon-8 F3	AATTTCCACCTCTGCCTTTAA	793	793
*FAM20B* exon-8 R3	AGATGAGTGGGCACATCAGG		
*FAM20B* exon-8 F4	TGACTTTGCACCTAAGTAAATTCTG	735	735
*FAM20B* exon-8 R4	GGTGGCTCATGCCTGTAATC		
*FAM20B* exon-8 F5	CTGAACCCATGATGTTGTATTA	666	666
*FAM20B* exon-8 R5	TCTTCCTATTGTCTCCTCCC		
*FAM20B* exon-8 F6	TTTTAAGGCTACTCAGTGTTGTG	842	842
*FAM20B* exon-8 R6	CTCCTGGATTCAAGTGATTCTCC		
*FAM20B* exon-8 F7	AGGCAAATCTTGGAGAAAAC	764	764
*FAM20B* exon-8 R7	TCTTGAATAATACTCTGAGCAAA		
*FAM20B* exon-8 F8	ATTTCCTGCCCTCCTCCAAC	879	879
*FAM20B* exon-8 R8	CTACCTTGTCACCACCCAGA		
*FAM20B* exon-8 F9	TAGTGTAAGGCTGCATTGTGG	765	765
*FAM20B* exon-8 R9	CTTGAGGAATTGAAGGGAAA		
*FAM20B* exon-8 F10	CAGCGAATAACTACTGAGCAA	514	514
*FAM20B* exon-8 R10	AAGGGAACTGAAATAGGAACCA		

### Statistics

The association between susceptibility to mesiodens and the genetic polymorphism of *PAX6*, *SOSTDC1*and *FAM20B* were assessed using IBM SPSS 20.0 software (IBM, Armonk, NY, USA). The data were analyzed using pearson chi-square test with theoretical frequency ≥ 5. For theoretical frequency less than 5 but at least 1 (≤20% cell), the data were analyzed by continuity correction. For the rest, Fisher’s Exact test was used. A *P*-value< 0.05 was considered statistically significant. The relationships between the characteristics of mesiodens and the polymorphisms with *P* value less than 0.05 were further analyzed using the same method described previously.

## Results

### Basic characteristics of patients with mesiodens

Four of the 50 patients with mesiodens (8%) patients had a family history of mesiodens. The basic characterizes of mesiodens are listed in Table 2.

**Table 2:** Characteristic of patients with mesiodens, mean ± SD, or n (%)

Numbers	50
Age (years)	11.8 ± 9.3
Gender	
Females	16 (32.00)
Males	34 (68.00)
Number of mesiodens per patient	
1	35 (70.00)
2	15 (30.00)
Growth status	
1 erupted	13 (26.00)
1 impacted	22 (44.00)
1 erupted and 1 impacted	8 (16.00)
2 erupted	2 (4.00)
2 impacted	5 (10.00)
Crown direction	
1 vertical	13 (26.00)
1 horizontal	4 (8.00)
1 inverted	17 (34.00)
1 inverted and 1 vertical	6 (12.00)
1 horizontal and 1 vertical	4 (8.00)
2 vertical	4 (8.00)
1 horizontal and 1 inverted	1 (2.00)
2 inverted	1 (2.00)
Family history	4 (8.00)
Located in maxilla	49 (98.00)
Located in mandible	1 (2.00)

### Associations between mesiodens formation and genetic polymorphisms

Considering the specific role of family history in mesiodens is still unknown, hence, careful family history is record in our study and excluded when we analyzed the association between mesiodens formation and gene polymorphisms. Removing patients with family history, three polymorphisms (rs750093295, rs667773 and rs3026393) were detected in *PAX6*. Two polymorphisms (rs6945425 and rs12699799) were detected in *SOSTDC1*. Twenty-nine polymorphisms (chr1:179025841, rs193196190, rs72707294, rs1024965514, rs745360443, rs778968805, rs2025584, rs140751029, rs9726948, rs16853612, rs9725887, rs9725888, rs4652352, rs147003645, rs72709441, rs4652353, rs4652354, rs56006430, rs3766625, rs3766626, rs775951319, rs16853619, rs2018786, rs16853621, rs188554154, rs530920451, rs9249, rs117216397, rs1220) were detected in FAM20B. Although, the T allele of *FAM20B* (rs3766626) appears to be associated with mesiodens after removing unqualified sequencing results (*P* = 0.051). There were no significant differences of *PAX6*/*SOSTDC1*/*FAM20B* gene polymorphisms between the two groups (Table 3). The distribution on genotype of these markers according to gender, the number of mesiodens, crown direction, and the eruption status are listed in Tables 4 and 5. The T allele of *FAM20B* (rs3766626) was associated with susceptibility to two mesiodens (*P <* 0.001; OR = 8.333; CI = 2.516–27.600).

**Table 3:** The gene polymorphisms in patients with mesiodens and controls

Marker	Gene polymorphism	Mesiodens	Controls	*P* value
rs2025584 (*FAM20B*)	AA/AG/GG	4/23/16	10/24/15	0.326
	A/G	31/55	44/54	0.223
rs140751029 (*FAM20B*)	CC/CT	40/4	43/4	1.000
	C/T	84/4	90/4	1.000
rs9726948 (*FAM20B*)	GG/GT	41/2	41/6	0.270
	G/T	84/2	88/6	0.282
rs16853612 (*FAM20B*)	AA/AG/GG	23/12/8	28/16/3	0.206
	A/G	58/28	72/22	0.171
rs9725887 (*FAM20B*)	CC/CT/TT	15/22/6	14/19/15	0.146
	C/T	52/34	47/49	0.120
rs9725888 (*FAM20B*)	CT/TT	2/41	6/42	0.273
	C/T	2/84	6/90	0.284
rs4652352 (*FAM20B*)	AA/AC/CC	5/15/23	6/12/30	0.584
	A/C	25/61	24/72	0.537
rs147003645 (*FAM20B*)	GG/AG	43/0	47/1	1.000
	G/A	86/0	95/1	1.000
rs72709441 (*FAM20B*)	CC/TT/CT	22/6/15	28/3/17	0.477
	C/T	59/27	73/23	0.262
rs4652353 (*FAM20B*)	GG/GT/TT	6/14/23	6/12/30	0.668
	G/T	26/60	24/72	0.430
rs4652354 (*FAM20B*)	CC/CT/TT	19/22/1	18/23/7	0.146
	C/T	60/24	59/37	0.159
rs56006430 (*FAM20B*)	CC/CG/GG	7/15/21	3/17/29	0.269
	C/G	29/57	23/75	0.123
rs3766626 (*FAM20B*)	GG/GT/TT	16/21/4	14/19/14	0.067
	G/T	53/29	47/47	0.051
rs3766625 (*FAM20B*)	AA/AG/GG/CC	6/15/20/0	2/17/27/1	0.277
	A/G/C	27/55/0	21/71/2	0.131
rs16853619 (*FAM20B*)	AG/GG	2/39	6/41	0.276
	A/G	2/80	6/88	0.287
rs72707294 (*FAM20B*)	GG/GC/CC	8/18/22	3/18/27	0.249
	G/C	34/62	24/72	0.116
rs2018786 (*FAM20B*)	AA/AG/GG	7/14/23	1/18/30	0.061
	A/G	28/60	20/78	0.076
rs16853621 (*FAM20B*)	AA/AG/GG	36/4/1	40/8/0	0.361
	A/G	76/6	88/8	0.802
rs188554154 (*FAM20B*)	GG/GT	43/0	47/1	1.000
	G/T	86/0	95/1	1.000
rs530920451 (*FAM20B*)	AA/AG	43/1	47/1	1.000
	A/G	87/1	95/1	1.000
rs775951319 (*FAM20B*)	CC	41	47	–
rs778968805 (*FAM20B*)	CC/CT	46/0	46/1	1.000
	C/T	92/0	93/1	1.000
rs745360443 (*FAM20B*)	GG/AG	47/1	49/0	0.495
	G/A	95/1	98/0	0.495
rs1024965514 (*FAM20B*)	CT/CC	1/47	0/48	1.000
	C/T	95/1	96/0	1.000
chr1:179025841 (*FAM20B*)	AA/AG	45/1	42/0	1.000
	A/G	91/1	84/0	1.000
rs193196190 (*FAM20B*)	GG	40	38	–
rs9249 (*FAM20B*)	AA/AG/GG	5/16/23	3/17/28	0.666
	A/G	26/62	23/73	0.392
rs117216397 (*FAM20B*)	AA/AG	42/2	48/0	0.226
	A/G	86/2	96/0	0.227
rs1220 (*FAM20B*)	AA/AC/CC/GG	20/19/4/1	17/23/8/0	0.428
	A/C/G	59/27/2	57/39/0	0.126
rs667773 (*PAX6*)	CC/CT/TT	27/13/2	36/10/1	0.451
	C/T	67/17	82/12	0.178
rs750093295 (*PAX6*)	CC/GG/CT	41/1/0	45/0/1	0.730
	C/T/G	82/0/2	91/1/0	0.226
rs3026393 (*PAX6*)	GG/GT/TT	6/26/10	17/20/10	0.056
	G/T	38/46	54/40	0.104
rs6945425 (*SOSTDC1*)	AG/GG	6/36	8/38	0.691
	A/G	6/78	8/84	0.704
rs12699799 (*SOSTDC1*)	AA/AG/GG	6/23/13	8/17/22	0.199
	A/G	35/49	33/61	0.369

**Table 4:** The distribution of AA genotype of FAM20B (rs2018786) according to eruption status

genotype	1 erupted	1 impacted	1 erupted + 1 impacted	2 erupted	2 impacted
AA	1	3	0	0	3
others	8	17	8	2	2

**Table 5:** The distribution of AA genotype of FAM20B (rs2018786) according to crown direction

genotype	1 vertical	1 horizontal	1 inverted	1 inverted + 1 vertical	1 horizontal + 1 vertical	2 vertical	1 horizontal+ 1 inverted	2 inverted
AA	2	0	2	0	2	0	1	0
others	8	3	14	5	2	4	0	1

## Discussion

A total of 8% of patients have a family history of mesiodens, which may indicate that the occurrence of mesiodens is partially determined by genetics. The patients with mesiodens were mostly concentrated in the northern and southern regions of Bengbu. The occurrence of mesiodens might have regional distribution characteristics.

*PAX6* is an important gene involved in a series of diseases including eye diseases, diabetes, autism spectrum disorder and mesiodens [14, 19–21]. Variants of *PAX6* are correlated with eye diseases and the insulin response [22–24]. Lei HH et al. identified that variants of rs667773 and rs3026393, and showed that the GG genotype of rs302693 was less prevalent in 20 patients with mesiodens than in 31 controls [18]. These results were further supported by our study. Polymorphisms in rs667773 and rs3026393 of *PAX6* were detected in the current study, and the mesiodens group might have fewer genotypes of GG (rs3026393) than do the controls. Polymorphisms related to other diseases were not detected in this study; however, this may be because the patients with mesiodens did not have any other diseases.

Mesiodens is the most common type among supernumerary teeth, and the development of supernumerary teeth is closely associated with bone morphogenetic protein (*BMP*) and Wnt signaling pathways [25]. *BMP* is required for *SHH* expression during early tooth development and postnatal root development [26]. However, *SOSTDC1* is an inhibitor of *BMP*, and the deletion of *SOSTDC1* in mice induces the formation of mesiodens [15]. *Wnt*, another signaling pathway, can be inhibited by *SOSTDC1*, located in the upstream of Sonic hedgehog (Shh), and induces the expression of Shh, followed by the induction of high *SOSTDC1* expression. Insufficient *SOSTDC1* enhances WNT signaling, which increases proliferation and continuous development of vestigial tooth buds and results in the formation of supernumerary teeth [27, 28]. In our study, two polymorphisms (rs6945425 and rs12699799) were detected in *SOSTDC1*, but none of them were found related to susceptibility to mesiodens.

FAM20B is a member of Family with sequence similarity 20 (Fam20) proteins containing FAM20A, FAM20B, and FAM20C in the human genome [29]. *FAM20A* knockout mice have biomineralization defects, and mutations in *FAM20A* have been found to be associated with amelogenesis imperfecta subsequently [30–32]. *FAM20B* null mice showed mesiodens [15]; however, the relationship between variants of *FAM20B* and mesiodens has not yet been reported. Our results suggest for the first time that individuals with T allele of *FAM20B* (rs3766626) appear to have a low risk of mesiodens, which was located in the 3′ untranslated region (3′ UTR) of corresponding gene. Although it isn’t translated into protein, previous and recent studies showed that variant in 3′ UTR region could impact the expression of mRNA [33, 34].

The current study provides information on the association between genetic polymorphisms and the occurrence of mesiodens; however, there are some limitations. The sample size (mainly the control size) and the number of genes analyzed in this study were limitations. The mechanism by which these polymorphism affect mesiodens is unknown. Further studies including more samples, more genes, and the mechanism of these polymorphism on mesiodens are needed.

## Conclusions

There were no significant differences of *PAX6*/*SOSTDC1*/*FAM20B* gene polymorphisms between the two groups. Further studies with large samples on T allele of *FAM20B* (rs3766626) are needed.

## Additional files

Additional file 1: The amplification sequences of *FAM20B*. (DOCX 22 kb)

Additional file 2: The amplification sequences of *SOSTDC1*. (DOCX 13 kb)

## Data Availability

The data and materials of the present study were available from the corresponding author.

## Abbreviations

AXIN2: Axin 2
BMP: Bone morphogenetic protein
ENAM: Enamelin
Fam20: Family with sequence similarity member 20
GREM2: Gremiln-2
PAX6: Paired box gene 6
SOSTDC 1: Sclerostin domain-containing 1
UTR: Untranslated region
