# Supplementary material for: Lack of association between PAX6/SOSTDC1/FAM20B gene polymorphisms and mesiodens
Source: BMC Oral Health. 2019 May 27;19:90. doi: 10.1186/s12903-019-0788-3 (PMC6537368; doi:10.1186/s12903-019-0788-3)
Supplement: Supplementary file 1 — The amplification sequences of FAM20B. (DOCX 22 kb) [file 12903_2019_788_MOESM1_ESM.docx]

| *FAM20B* exon-1 F | GTCCTGCTGCTTGGCTGCCTACCTAC |
| --- | --- |
| *FAM20B* exon-1 R | CCTTCAGCCGCGACCGCACA |

GTCCTGCTGCTTGGCTGCCTACCTACCTAA

GAAAAGCAGGAAAGAAAAAGAGAAGACGGGGTGGGGGATGGAGGACAAATACAGAAAAAG

AAAAAAGAGCGAAGGAAGTGAGAAGTGAGAGGCCAGTTTATGTAAGCGGGCAGGGCGGCG

CGGCCGGCAGGAGACCGGCGCCGGCGCCGAGGGAGTTAAGCGGACGGGCCCGGTATGGAA

GAGGTTAAGGGGGGCAGTCCGGAGCGGGTCCGGTAGGGGCCGCCTACTGGGCGGGGGCGG

GGCCGCGCCGAGCGAGCGCTGGGATTGGCCGTCGGGCAGGCGCTACGCTAGGACGGTACC

AACAGCCGGGGGCCTCGGAGGCGGGGGGGTGGGTGTGGGACTGCCGCTCTGCGCGGCGAG

AGGTGGCCTGGGAATGGCCGGGCCGGGGGTGGGCCGGAGCCGCTGTGGCGGCGGCGGCGG

CTGGGGGCGGTGAGCGCGGCGTGGGGCTGCCCCTCCCCGGAGGCGGCGGGGGCGGCCGGG

GCCGCGCCGCACCGCACCGCGCGGGCGGCCATGGAGCGAGCCTAGGGCCCGACAGGTGAG

TGGCGCGGGGGCGGGGGAGGGCGCGCGACCCCCGCCCCCGGCGCGGCGGGCGGGAAAATG

CGGACCGGCACGCGGGCGGGGCACGGTACGTAGCGCGCGCCGGGCTGGGGCCGCCTGGGG

GCGGGGCGCCTCCCTGAGGGGCCGGGCACCGGGTGCCCTGAGCCGTCCCCGAGTGGCTGC

GGGCGGGTCCCGGGCGAGGGCCCGAGGCTTCCTGGGAGACCCGCTCGGGCCGCGTGTCCC

TGTGTGCGGTCGCGGCTGAAGG

| *FAM20B* exon-2 F | ACTGCTGCCATCATTAGGTCC |
| --- | --- |
| *FAM20B* exon-2 R | CAGTGGGTTACCAGGTGTTCT |

ACTGCTGCCATCATTAGGTCCTTC

TCCAGATTAGTTAATTTTAAATTATTGAACCTTCTTGAGCTCAAGAATAGAAAGCCTAAA

AGGGTGAGTCATATATTAGAGGCTTACAAAAGATTCAGGGGTGGAAGGACAGAATTTTTG

ATCAAATTTTGCTTTGTACTTGGGCTTGCAGGAACTGTGGAAGGTGCATCAGTGAAGAAA

TGGACCAATGTGTATAATCATGGAATCTCCTTGCTAACCATCACCACCAGCTCTCCTTAA

TACATGAGCAAGAGTGGGTCAGGGGAGAAGGAAAAGAGGTCAACATGAAGCTAAAGCAGC

GAGTCGTGCTGTTAGCAATTCTCCTTGTCATTTTTATCTTCACCAAAGTTTTCCTGATTG

ACAACTTAGATACATCAGCTGCCAACCGGGAGGACCAGAGGGCCTTTCACCGAATGATGA

CTGGCTTGCGGGTGGAGCTGGCACCCAAGCTGGACCATACCTTGCAGTCTCCCTGGGAGA

TTGCAGCCCAGTGGGTGGTTCCCCGGGAAGTGTACCCTGAAGAGACACCAGAGCTGGGGG

CAGTCATGCATGCCATGGCCACCAAGAAAATCATTAAAGCTGATGTGGGTTATAAAGGGA

CACAGCTGAAAGCCTTACTGATACTTGAAGGAGGCCAGAAAGTTGTTTTCAAACCTAAGC

GGTAAGTTTTGATCTTGGAAGCTGCATGTGCTAGTTGGTTGATTCATTTAACTTGGGATT

TATATAAGATTTATTTTGTCATCTTCTCTTGGAAGTCTCTTCAGTAAAATAAGAGGGGTA

GACTAGATCTCTAAAGTCTTTTCTAGCACTTAACATGAATCTACCTTTATAGTATCATAT

AGAATAGTCTTCCTGCCCTAAAAATCCTCTGTGTTCTACCTACTCATTTTTCCCTCTCTG

CCCCAGAACACCTGGTAACCCACTG

| *FAM20B* exon-3 F | AATCAGGCTTGCTAATGGGTG |
| --- | --- |
| *FAM20B* exon-3 R | AGGCCAGAAATGAAATGACCTA |

AATCAGGCTTGCTAATGGGTGTATTCTACCACTTGTGCTGT

CACCTGTTACTGGTGTAATCCACGTATACATCTGTGCTTCCCATTCACTTTGCAGGTATA

GCCGAGACCATGTGGTGGAAGGGGAACCGTATGCTGGTTATGATAGACACAATGCAGAGG

TAGCAGCCTTTCACTTGGACAGGTGCGTATGATCACAGCAGCTTATGTTCATTTTGTTTG

CTTTCAAAAATCTTTCTTGGAGAGGACTCGTGGACTCCTTCAGAAGGATGCAACACTAAT

AAATAACTTGTTATCGATTTTAGATAGCTAATTGACTAGTTCCATTAAACTTGTATGAGT

CTCATAATTTGGTTGGCACCAGGAATACTGAAGTATTTTACTTCCATGCTTCTTTAGGTC

ATTTCATTTCTGGCCT

| *FAM20B* exon-4 F | TTAATTTGCTCTGTGGGCTTAG |
| --- | --- |
| *FAM20B* exon-4 R | CACCTGCTTTCACCATCACTA |

TTAATTTGCTCTGTGGGCTTAGCCTTTTAGATCAGTCTGCTTTTTTTTTTTTAAC

CAACATGCTGTTTTATTTAAAGATTTGAACCCATATATGAGTTTGGGTCCCATTTTTTTT

CTAATACTTTCCTAAAATCTATTTTTAATTTTTATTATGAAAAAATTTCTATCATACAAA

AGTAGAAAGAATAGTATAATGAGCCCATATACCCACTCTGTAGATTTAAAAAGTGTTCAC

ATTTTATCAGATTTGTCTTATTCTTTTTGCTGAAATGTATATTAAAATAAATTATACACA

CCTTCACATTAAATTCCTAAATATATGTACCTCTAAAAAATTAGGTTTAGCTACTAGCTA

AAAACCCTTTACACATTTGACATGATGAACTGTAATTTTTAAGACTGTTACTTAAAAAAC

ATCCCCTAAGATTTTTCTCTTCTGTTCTTCAATCTTCCAAACCAGGATTCTGGGTTTCCA

CCGAGCCCCCTTGGTAGTTGGCAGATTTGTTAATCTTCGGACAGAGATCAAACCTGTCGC

CACAGAGCAGCTGTTGAGCACCTTCCTAACTGTAGGTAAGAAGATTGTAGAGGACATTTA

TATAGGGGAATGATTAATAAGTTAAAATGGGGCATTGTTGAGCAAGCTGGCTCATGACTT

TTAGGAATTGAAGAACTCAGTGGAATACAAAAGCAAATCAGACAACCAGCTCTCAGGTTA

TTTGGGAAAATGAATCTAAAGGAGATGGCAGGAAATTGAAATTTTCCTGTATACTTTGTG

TTGTCTGTGACATAGGGAGTGAGGTCATTTAGTGATGGTGAAAGCAGGTG

| *FAM20B* exon 5-6 F | AGAGTGAGACTGGGTAGAAAGGA |
| --- | --- |
| *FAM20B* exon 5-6 R | TAGCCAAGAAAGAACGATGTAG |

AGAGTGAGACTGGGTAGAAAGGACACAGGAGGCAAGGGCAACTTACTTTTGGGTGTATAT

CCTTTTGTACTTTTTAAATTTTTTTTACACCGTGTGCTTTTTTATTACCTATACAGGAAA

ATAAATGTTTGAAATATAGTAAACTCAGGGTCTTTGTTTTCACCATGCTTATTTACTTAG

CTACTCTGTTCTGCATTATTTGGGAGAGAACTTGGAGTCTTCGTTATTTCCTTAAGTGTA

TTTGGCTCCTTTCTGTCCTTTAGGAAACAATACTTGTTTTTATGGGAAGTGCTATTACTG

CCGAGAAACAGAACCAGCTTGTGCTGATGGAGACATAATGGAGGGATCTGTCACACTTTG

GCTTCCAGATGTGTGGCCTCTGCAGAAGCACCGTCACCCATGGGGCAGGACTTACCGAGA

AGGCAAATTGGCCAGGTAAATGCTCCTATGAGCCATTACTTAATTCTCCCCTGTGCCTAG

CCAGGTGCCAACTCTGTAAAGGAGCCAGCAGTTCTGTCCAAGAGTGAAAAGAACTGTGGA

GTCAGGGGCAAACCGGTAGGTAGGAACTCTGTCTTCTCTTTGTGTGTAACAGTGCCAGGT

ACAGTGTTGTCACTCAGTGCTGTGATGCTGCTTGTCTCCAGGTGGGAGTATGATGAGAGC

TACTGTGATGCTGTGAAGAAAACGTCCCCTTATGACTCTGGCCCGCGCCTCTTGGACATC

ATTGACACAGCTGTCTTTGATTACCTGATTGGCAATGCTGACCGCCATCACTATGAGAGC

TTTCAAGATGATGAAGGCGCTAGTATGCTCATCCTTCTTGATAATGCCAAAAGGTGAGAC

CAGCAGGACTGTCCTTGTCAGGGAGGGGTTTCTGTATATGAAAGAAGGGCATTTTCCAGA

GCATCCTGGAGAATATCCAGAATGCAATTGATAGGCAACATCCTTCTTTTTTCCACTTGG

AGTCTTTGCTATTGGTACCTGTCCTTCTCTCCACTCTCAGGGATCTCAGTACTTCAAAAA

ATGGAAAAGTGGTTCTTCCAAGGAGTCTAAGACTCTATGAGCCTCTTCTCAGACAACTGG

AAAACCAGGGTTATTCATTTGTTACTACCTACATCGTTCTTTCTTGGCTA

| *FAM20B* exon -7 F | AAGTTCTCCCTTTGGTCTGTG |
| --- | --- |
| *FAM20B* exon-7 R | TTTGGGTTATCTGCCTTCAC |

AAGTTCTCCCTTTGGTCTGTGAACTCCGCACTGTGTTTACCATAGTTATGCTCCAGC

ACCTTGTGATATCTTGGCAGGAAAATGTCCCTTGTAAATGAGTATATTTGTTGTCAGATA

TTTTAGTACAGGCTGTTAGGGATCCTGTGTCTAATTGGTCATGCCCCGCTTGTTTTCAAT

TTAAGTGATTTGAGCTGCTGGAGCGTGAAATTATCTAGGATCACATTTGAGTTTCATATA

GAATTTGCTATTGCTTTGATGCTAAGAAGAGAACAGTACTTCCACCTAATTCACTAAGTT

TTAATTTAATTTTCAGCTTTGGGAACCCCTCGCTGGATGAAAGAAGCATTCTTGCCCCTC

TCTATCAGTGTTGCATGTAAGTTATGCACAGCAAATACATGTGCCTGCATTGCCTTCTTT

TCCAGGCTCAGGTAACAGTACATCCATTTTCTTGGCCCTCAAACTTTCTGATACCTGAGC

AGTGCTTTTCATACTTTTCCAACCTAGAACCTCTGGACATCCTGAAATGGGATATCAGGG

AAAGGTGAAGGCAGATAACCCAAA

| *FAM20B* exon-8 F1 | CCATAATTTAACTATTTCCCAGTCG |
| --- | --- |
| *FAM20B* exon-8 R1 | CCAATCCCAGTATTCATCTATCC |

CCATAATTTAACTATTTCCCAGTCGTTAGACATT

CAGATTGTTCAACATTTTTCCTGTTATAAACAGCACTGCAGTGAACATGTGTGTAAATCT

CTGTATATATCTCTGATTAGTTACACAGAAACAATATCAAAACATAGTAATGTGGCTTAC

TCATTATGTAGGGGTAATTCTAAAGCAAAAATTATTTAGTTAAAGGATTTTACCTAATTA

TTTTTTCACTTTTGTCTGCCTAGCAGGCTTTCAACTCCGTTTGATAATGAGAAGTTTTAT

GTACCTTGTTCTATAACTTTTTCTCCCAGCATTCGGGTGTCCACCTGGAACAGACTGAAC

TACCTAAAGAATGGTGTGCTAAAGTCTGCCTTAAAATCTGCCATGGCCCATGACCCCATC

TCCCCAGTGCTCTCTGATCCTCATCTGGACGCCGTGGACCAGCGGCTCCTGAGTGTCCTG

GCCACCGTGAAGCAGTGCACCGACCAGTTTGGGATGGACACAGTACTGGTGGAAGACAGG

ATGCCTCTCTCACACTTGTAATTCTCGACACAAAATAAGTGAAACTTCTTTTTACAAAGA

TAGAGAAACAGCACAATCAATTCCAAATGGTATGAGATGGATTGGAAGTGGCCAGCAGCA

AGTTCTGGTGACGGGACAGAGTGGCCTTGGATGTCTTTGGTATTTTCTGTAGTAGAAACT

AAAGCAAAGACCACAAGTTTCAGAGCATGGAGACATTCCTGCTGAATCGCCTTCTCACCT

CCTCGGCAATTGCTCATTCTAGGGTTGGGCATCATAGTTGGTCAGTCTTAATTCCCATGC

CAAAGGACAAACAGGTGTGACATTT**GGATAGATGAATACTGGGATTGG**

| *FAM20B* exon-8 F2 | TGGTGACGGGACAGAGTGGC |
| --- | --- |
| *FAM20B* exon-8 R2 | CAGTTTGCTTTGTTAATTTGGGAAG |

**CTGGTGACGGGACAGAGTGGC**CTTGGATGTCTTTGGTATTTTCTGTAGTAGAAACT

AAAGCAAAGACCACAAGTTTCAGAGCATGGAGACATTCCTGCTGAATCGCCTTCTCACCT

CCTCGGCAATTGCTCATTCTAGGGTTGGGCATCATAGTTGGTCAGTCTTAATTCCCATGC

CAAAGGACAAACAGGTGTGACATTTGGATAGATGAATACTGGGATTGGCTCTGGAGCATG

TGTTTTGAGTTGAACCTTGCAGTCCTTTCTCTACGCCCGTGGATTTTGTGGAAACACTTT

GCAATCTCTTTGTCTTTTTTTTTTTTACCAGAACTAGTTACATTGGAATGCTTACTGTCC

TACAGAGTGGCAGCAAATAAAACCTTGCATTCCATCAAGCCAAAATAGCACACTCTGTTA

GAGGAGATACATGTTTAAGATAGAATTGGAGGGAAGGACAAAAACAGAAAAATGTTTGGG

CTTTTAAGCCATTGGGTAGTATTGTTTTGATGATCTTAGAGGAGGGAAGAAGAGAGAGAG

ACCCAATGGTAGAACCAGAATCAGGGAGATGACTGAACTACTGAAAAACAGGTTCCCTTG

TATTTAGGATCTTAAGGTGTATAAAAAGCAAACATGACTTTGCACCTAAGTAAATTCTGC

ATTCTCATAGTTGTGTCCCAATTAACCAAAAAGTTGTCTCTAGAGAAAATACTATTACAA

TCTAAGCATGATTCTCTGTGGAGACTAATTTTTTCCCCTTTTGCCAAAAGCAGTC**CTTCC**

**CAAATTAACAAAGCAAACTG**

| *FAM20B* exon-8 F3 | AATTTCCACCTCTGCCTTTAA |
| --- | --- |
| *FAM20B* exon-8 R3 | AGATGAGTGGGCACATCAGG |

**AATTTCCACCTCTGCCTTTAA**GGCATTTTTGTCACTG

AAGCTGCTGTTCCCAAGAGATCGGCAACCTTTTTGTCCCTTTCTCATAAGAAAGGGACAC

TCCTACAGGTGAGAGTGTATACCTTACTCTCTCAGATAAGTGGCTGGACTTATCTTGTGA

TTTGGGGCCATGGAAGATTGGAAACAAAGATTTTAAGCCTTCTTCTTTTTTTCTTTTTTC

TTTTTTTTTTGAGACCAAGTCTCACTCTGTTGCCCAGGCTGGAGTGCAGTGGCACGATCT

TGGCTTACTGCAACCTCCGTCTCCCAGGTTCAAGCGATTCTCTTGCCTCAGCCTCCAGAG

TAGCTGGGATTACAGGCGCCCGCCATCGTGCCCAGCTAATTTTTATATTTTTAGTGGAGA

CAGGGTTTCGGGTTTCACCATGTTGGCCAGGTTGATCTTGGACTCCTGACCCCAGGTGAT

CCACCTGCCTCAGCCTTCCAAAGTGCTGGGATTACAGGCATGAGCCACCGTGGCCGGCCA

AGATTTTAAGCCTTCTGAGCCTTGAAATTGAGGAGGTTAAAAGGAAGAGCCTTAAGATTT

TGATTTATGTCAAATCCTAATTCTATCATTCAGTCTTGTTTGGAGTTCTGAACCCATGAT

GTTGTATTATGCTTCTTTCTCCTCTTAGCACTCTCAAATTTCAGGTTTGTAAAACACAGT

TTTTGTTTTGTGTTCTGGCAAAGTGATCTCAACATGTAAGTAGTTGCAGTAAAACACAGG

GGCAAAGGAAGACAGG**CCTGATGTGCCCACTCATCT**

| *FAM20B* exon-8 F4 | TGACTTTGCACCTAAGTAAATTCTG |
| --- | --- |
| *FAM20B* exon-8 R4 | GGTGGCTCATGCCTGTAATC |

**TGACTTTGCACCTAAGTAAATTCTG**C

ATTCTCATAGTTGTGTCCCAATTAACCAAAAAGTTGTCTCTAGAGAAAATACTATTACAA

TCTAAGCATGATTCTCTGTGGAGACTAATTTTTTCCCCTTTTGCCAAAAGCAGTCCTTCC

CAAATTAACAAAGCAAACTGAAATAATACCTTGAATAACAGGTTGCCTGTGGTCTCTGTC

ATCCTCGTTTCTCTTCTGAAATGAATTTCCACCTCTGCCTTTAAGGCATTTTTGTCACTG

AAGCTGCTGTTCCCAAGAGATCGGCAACCTTTTTGTCCCTTTCTCATAAGAAAGGGACAC

TCCTACAGGTGAGAGTGTATACCTTACTCTCTCAGATAAGTGGCTGGACTTATCTTGTGA

TTTGGGGCCATGGAAGATTGGAAACAAAGATTTTAAGCCTTCTTCTTTTTTTCTTTTTTC

TTTTTTTTTTGAGACCAAGTCTCACTCTGTTGCCCAGGCTGGAGTGCAGTGGCACGATCT

TGGCTTACTGCAACCTCCGTCTCCCAGGTTCAAGCGATTCTCTTGCCTCAGCCTCCAGAG

TAGCTGGGATTACAGGCGCCCGCCATCGTGCCCAGCTAATTTTTATATTTTTAGTGGAGA

CAGGGTTTCGGGTTTCACCATGTTGGCCAGGTTGATCTTGGACTCCTGACCCCAGGTGAT

CCACCTGCCTCAGCCTTCCAAAGTGCTGG**GATTACAGGCATGAGCCACC**

| *FAM20B* exon-8 F5 | CTGAACCCATGATGTTGTATTA |
| --- | --- |
| *FAM20B* exon-8 R5 | TCTTCCTATTGTCTCCTCCC |

**CTGAACCCATGAT**

**GTTGTATTA**TGCTTCTTTCTCCTCTTAGCACTCTCAAATTTCAGGTTTGTAAAACACAGT

TTTTGTTTTGTGTTCTGGCAAAGTGATCTCAACATGTAAGTAGTTGCAGTAAAACACAGG

GGCAAAGGAAGACAGGCCTGATGTGCCCACTCATCTATGGACTCAGAGCTGTGTGCTTTG

CTCCTGCATCTTGTTGAGGTGCTGTTCCAGCTTTGCATTTCTGTCAAGTAGAGGCGAATA

TATAAACAGTGTGGTTGAATACATTTAATGCCAGCCATTGGAAACTAGTTTTAGGCAACC

ACTCTCAAAAACAGCTTTAGAATTTATGCCCAGTTTTCTTGCATTGAAAGATAACTGAGT

AATAACCTGTAACTATTTTTAAATGGCATGAAATTAGGAAACTTTTGTACATTTTATATA

CATTTTGAGATGAACAGAACAATGGGCTGAGTTATAAAAAGCGTGTATTGAATTTAAGAA

GACAGACTAGCACAAAACACAGAATTCGTGTTAACCAAAGGAGGCATTGATTTCAGTTTT

AAGGCTACTCAGTGTTGTGTGTCCAGGGAAATTCACAGCTCAGTATGAGAATACCTTGGT

TAGTGCTCACCCACAAGCTTCCAGGAGCCAGCT**GGGAGGAGACAATAGGAAGA**

| *FAM20B* exon-8 F6 | TTTTAAGGCTACTCAGTGTTGTG |
| --- | --- |
| *FAM20B* exon-8 R6 | CTCCTGGATTCAAGTGATTCTCC |

**TTTT**

**AAGGCTACTCAGTGTTGTG**TGTCCAGGGAAATTCACAGCTCAGTATGAGAATACCTTGGT

TAGTGCTCACCCACAAGCTTCCAGGAGCCAGCTGGGAGGAGACAATAGGAAGAGATGTCA

TCTCTGCTCTCCCTGTAAATGTTAGTTGAACTAAGTTATGGATTTGTGGTCTTTCAAATA

CATGACGCCTTTAGTATGCCACACTGAAATGAATAAGAAGTCTTCTGAAACTGGGAACTT

CATAACATTGAAGGCAGAAGATTCTGCTAAGGAAAAAAGCAGGCAGGAAAGAAAATGTCT

CATCCTTTCTTGAAAGCATTTGCAGAAAATATATCATTTCATTTTATTCCCATCTGTTTT

CAAACTCGTGATCTTAAAAGGCATTCTGATGATAAATTTAGAATTTTCATCTATAAAATT

TAGAACTCTAATCCATAAAGTTAGAATTGAGCTAATAGAGTGGTATGACATGGCACTAAA

AATATAAATTTTTGTTGTAAGTCAGGATTGGAGTAAGCTGGAAAAGTATGTTTAGGCAAA

TCTTGGAGAAAACCAACCATAAACTTACAGCTCTAAAATTCAGAAAGCCCTAAAATTTCA

AACACTGTTTGAAAGAAGAGGTGGGGGCCGGGTGCCGTGGCTCATGCCTGTCATCCCAGC

ATTTGGGAGGCTGAGGCAGGCAGATCACCTGAGGCCAGGAGTTCGAGACCAGCCTGGCTG

GCTAGCATGGTGAGACCGTCTCTACTAAAAATGCAAAAATTAACAGGGCACGGTGGCATG

CGCCTGTAGTCCCAGCTACTCGGGAGGCTGAGGCA**GGAGAATCACTTGAATCCAGGAG**

| *FAM20B* exon-8 F7 | AGGCAAATCTTGGAGAAAAC |
| --- | --- |
| *FAM20B* exon-8 R7 | TCTTGAATAATACTCTGAGCAAA |

**AGGCAAA**

**TCTTGGAGAAAAC**CAACCATAAACTTACAGCTCTAAAATTCAGAAAGCCCTAAAATTTCA

AACACTGTTTGAAAGAAGAGGTGGGGGCCGGGTGCCGTGGCTCATGCCTGTCATCCCAGC

ATTTGGGAGGCTGAGGCAGGCAGATCACCTGAGGCCAGGAGTTCGAGACCAGCCTGGCTG

GCTAGCATGGTGAGACCGTCTCTACTAAAAATGCAAAAATTAACAGGGCACGGTGGCATG

CGCCTGTAGTCCCAGCTACTCGGGAGGCTGAGGCAGGAGAATCACTTGAATCCAGGAGGC

GAAGGTTGCAGTGAGCTGAGATTGTGCTGCTGCACTCCAGCCTGGGAGACAGAGCGAGAC

TCTGTCTTAAAAAAAAAAAAAGGAGGTGAATTTTTTTTTAAGTTTTGTAACACTGTCCTA

CTTTATTTATTAGAATCTAAGGCTGTTACAATCAAGTCGTTGCAGGGTTTGGATCAGCTG

TAAGTTAGGTATGCCTACCAAACATCCAAAGGTAGACGTGGAGACATTTTAATACTACAA

AACTAGGAAAATCAGAACTCATGGCCATTTCCTGCCCTCCTCCAACTTGTTAAAACATGT

TTATTCTAAAGTTCGAATGGATAAATTTGAGTATAAAGGTTTTGTTATAAAACTGTTCTT

TAGTGTAAGGCTGCATTGTGGGTTTGGGGGAAATGTAAATAATTTTCTGTGTAAAACAAA

TTCATAGGATCTGA**TTTGCTCAGAGTATTATTCAAGA**

| *FAM20B* exon-8 F8 | ATTTCCTGCCCTCCTCCAAC |
| --- | --- |
| *FAM20B* exon-8 R8 | CTACCTTGTCACCACCCAGA |

**ATTTCCTGCCCTCCTCCAAC**TTGTTAAAACATGT

TTATTCTAAAGTTCGAATGGATAAATTTGAGTATAAAGGTTTTGTTATAAAACTGTTCTT

TAGTGTAAGGCTGCATTGTGGGTTTGGGGGAAATGTAAATAATTTTCTGTGTAAAACAAA

TTCATAGGATCTGATTTGCTCAGAGTATTATTCAAGAATGTATTAATAAGGCATTGCCCC

CTGTTTGCACTCAGGGTTAATATGTCAAATGAAATTTAAGAAGGAAATGGAAGAATTCAG

GTACATTAATTGCATATTATTTTGGGAAAGATGAGTCCTATACGTGGCAATTTTTCAATG

TCATCTGAAGCCAGCATTATCTTCCAAAGAAATCGATCTTTTTTTTCTAAAAAAAAAAAA

TGCTTTTGCCTTCCCTTCCCTTCCCATCCGCCATATTTCTTCAGCCTTTCTTCTCGATCA

CCCGTGTATTCTTTGACCAGTAAATGACCACACCTCAATGATGGTAAAACAGCATCATCA

GTAAGCTATCTTATATGCCTCATCCTGTGAGTTTGAGCTTCAGGAAACATGAGTAAAAGT

ATATGTAATGTATATAGTCGTATATGTATTCTAGCAAGAAAAACATATTTATTTTGACAA

AGGGGAACACTGACTTTCTGAAGGATTCAGAAAGAACCTTAGTGAAAGGTTCTCAGTCTC

TGAGAGTGGACCCTAATTAACATAAAGACCATTCATCAGCGAATAACTACTGAGCAACTC

TAGTGTGCCAGCACAGGCCAGACATACTAGTGAGCCAGGCACATCTGGCCTTGGGAAACT

CATCCTACAGGGGAAGGCCAGTTTTTTTCCCTTCAATTCCTCAAG**TCTGGGTGGTGACAA**

**GGTAG**

| *FAM20B* exon-8 F9 | TAGTGTAAGGCTGCATTGTGG |
| --- | --- |
| *FAM20B* exon-8 R9 | CTTGAGGAATTGAAGGGAAA |

**TAGTGTAAGGCTGCATTGTGGG**TTTGGGGGAAATGTAAATAATTTTCTGTGTAAAACAAA

TTCATAGGATCTGATTTGCTCAGAGTATTATTCAAGAATGTATTAATAAGGCATTGCCCC

CTGTTTGCACTCAGGGTTAATATGTCAAATGAAATTTAAGAAGGAAATGGAAGAATTCAG

GTACATTAATTGCATATTATTTTGGGAAAGATGAGTCCTATACGTGGCAATTTTTCAATG

TCATCTGAAGCCAGCATTATCTTCCAAAGAAATCGATCTTTTTTTTCTAAAAAAAAAAAA

TGCTTTTGCCTTCCCTTCCCTTCCCATCCGCCATATTTCTTCAGCCTTTCTTCTCGATCA

CCCGTGTATTCTTTGACCAGTAAATGACCACACCTCAATGATGGTAAAACAGCATCATCA

GTAAGCTATCTTATATGCCTCATCCTGTGAGTTTGAGCTTCAGGAAACATGAGTAAAAGT

ATATGTAATGTATATAGTCGTATATGTATTCTAGCAAGAAAAACATATTTATTTTGACAA

AGGGGAACACTGACTTTCTGAAGGATTCAGAAAGAACCTTAGTGAAAGGTTCTCAGTCTC

TGAGAGTGGACCCTAATTAACATAAAGACCATTCATCAGCGAATAACTACTGAGCAACTC

TAGTGTGCCAGCACAGGCCAGACATACTAGTGAGCCAGGCACATCTGGCCTTGGGAAACT

CATCCTACAGGGGAAGGCCAGTTTT**TTTCCCTTCAATTCCTCAAG**

| *FAM20B* exon-8 F10 | CAGCGAATAACTACTGAGCAA |
| --- | --- |
| *FAM20B* exon-8 R10 | AAGGGAACTGAAATAGGAACCA |

**CAGCGAATAACTACTGAGCAA**CTC

TAGTGTGCCAGCACAGGCCAGACATACTAGTGAGCCAGGCACATCTGGCCTTGGGAAACT

CATCCTACAGGGGAAGGCCAGTTTTTTTCCCTTCAATTCCTCAAGTCTGGGTGGTGACAA

GGTAGGGGCTAGGTACTGGACTACCACAGGTTTTTAGGAACTAAGGTGTTTCTCATAAAC

ACAAAATGTTGGGTGAAACTGGGAACAACTACTCAGAAGCTCATTTATTTGCTTAAATGG

AAAGTGTGGGAGCCACTACCCTCTCTTTTGATCTGCCAAGGATTTCCTCTCAGAGCTGTT

GCACAGACAGAGATTGTACTTGGTAAGATACCAAACAAGACAGATATGGATCTAAATTTC

TAATGTGTTCTATGGGTTTCAATTCTGAAAAAAGAAAATGAATAAAGATTTTAATAAATA

TTGATATCTGATCTTTTCCTCTTCTTTCCCCCTTCAGGATATTTCCAGTGGTTCCTATTT

CAGTTCCCTT
