# Supplementary material for: Lack of association between PAX6/SOSTDC1/FAM20B gene polymorphisms and mesiodens
Source: BMC Oral Health. 2019 May 27;19:90. doi: 10.1186/s12903-019-0788-3 (PMC6537368; doi:10.1186/s12903-019-0788-3)
Supplement: Supplementary file 2 — The amplification sequences of SOSTDC1. (DOCX 13 kb) [file 12903_2019_788_MOESM2_ESM.docx]

| *SOSTDC1* exon-1 F | ACAAGTGATGAAGTCCAACTCT |
| --- | --- |
| *SOSTDC1* exon-1 R | TGTGAGCTAATGCTACCAGAA |

ACAAGTGATGAAGTCCAACTCTGAAATTTCAGGCA

ATTTGTATACCAAGCTCCTCCTTTTCTGCAGTCTTCTTTTCCATTGGTAAAATTCTTTTG

CAGGGTCATGTAGGGATCCCACCCCTTCTCTGTGTTTTCACTCTGAAGCTCTACACAACT

TTACACCTGAATGAACGCCAAACCTCTATGGATATATAAAGGGAAGCTTGAGGAGGAATT

TCACAGTTACAGTGCAGAAGCAGAAGCAAAAGAATTAACCAGCTCTTCAGTCAAGCAAAT

CCTCTACTCACCATGCTTCCTCCTGCCATTCATTTCTATCTCCTTCCCCTTGCATGCATC

CTAATGAAAAGCTGTTTGGCTTTTAAAAATGATGCCACAGAAATCCTTTATTCACATGTG

GTTAAACCTGTTCCAGCACACCCCAGCAGCAACAGCACGTTGAATCAAGCCAGAAATGGA

GGCAGGCATTTCAGTAACACTGGACTGGATCGGAACAGTAAGTGTGTTTTACTTGTACAG

TTTTTTTTTTTCTTTTCTGGTAGCATTAGCTCACA

| *SOSTDC1* exon-2 F1 | TGAAAGTGTCCCTATACTATCC |
| --- | --- |
| *SOSTDC1* exon-2 R1 | AACTACAGGATACGTGGAAT |

TGAAAGTGTCCCTATACTATCCAGACAGATTTTTTTCACTGT

GAAAATAAAGTAGGTGCTCACTTTCTATTTGGCAATTTGCTATCATTTGCCTATTATTTT

TGTCATTGCAGATCACTTTTTAAAAGGTCTTCATTTGCATTTTTTCTCTGATGCACATTC

TTTTTTGTTTCCTGCAGCTCGGGTTCAAGTGGGTTGCCGGGAACTGCGTTCCACCAAATA

CATCTCTGATGGCCAGTGCACCAGCATCAGCCCTCTGAAGGAGCTGGTGTGTGCTGGCGA

GTGCTTGCCCCTGCCAGTGCTCCCTAACTGGATTGGAGGAGGCTATGGAACAAAGTACTG

GAGCAGGAGGAGCTCCCAGGAGTGGCGGTGTGTCAATGACAAAACCCGTACCCAGAGAAT

CCAGCTGCAGTGCCAAGATGGCAGCACACGCACCTACAAAATCACAGTAGTCACTGCCTG

CAAGTGCAAGAGGTACACCCGGCAGCACAACGAGTCCAGTCACAACTTTGAGAGCATGTC

ACCTGCCAAGCCAGTCCAGCATCACAGAGAGCGGAAAAGAGCCAGCAAATCCAGCAAGCA

CAGCATGAGTTAGAACTCAGACTCCCATAACTAGACTTACTAGTAACCATCTGCTTTACA

GATTTGATTGCTTGGAAGACTCAAGCCTGCCACTGCTGTTTTCTCACTTGAAAGTATATG

CTTTCTGCTTTGATCAAACCCAGCAAGCTGTCTTAAGTATCAGGACCTTCTTTGGGAATA

GTTTTTCCTTTTCAAGTTTTTCAAGATGTAGGTATATCCATGAATGCAATTTGCATTTAA

ATTCCACGTATCCTGTAGTT

| *SOSTDC1* exon-2 F2 | AAATTCCACGTATCCTGTAG |
| --- | --- |
| *SOSTDC1* exon-2 R2 | CATGTTAGAGGCAACAACA |

**AA**

**ATTCCACGTATCCTGTAG**TTTTAATTCCTCATTGTTCTTAAAAGACTGTTGATACTATAA

ACATCAGTGAATCATTATATTTTAAAACAGAAAAGGGCTTCTCAGATACCCTCCATCTAC

TGGCCCATCCCCTCTCCTAAACAAAACTCCTTCAAAACAGGTTAAAAAAAATATGTTGTC

ATGAATCTTCACAGTAACATTTCAGAAAGGTGCTTTTTTGGTACTCTTCATGGGAACAGT

TTAGCAGCCATGAGTGATCTTCCTTTGAAAGAGAATGAAAGACCCTGTGACATTTCACTT

CAAAAATAAGCCCTGTAGCTCTTTACGGTCGCATAGTATGAAATTATACCCTGCATGCTG

ACCCTCGCTTGGAATGGAATGCCAGAAATGCATGGCAGCAGCTAATAAGTAAAGCTGATT

AACTATTTATTTGTCAATGTTATTATTTAATGAGCTTTCACATGTGATTTGTTTCAAAAC

TTTAATTTTTTAATGTTTTGAAACTTTTTCATGGACCTAAATATTTTCCTATATGATTTG

TGGTTGATTAGAAATATGAAATACATGTTGTAGATATGTAAAATGAATATTTTAGTCTCC

TTATTACATATATGTTCATGGTGAACTTTATCAATAGTATGGATCTTTTTAAATCAATAA

GATGCTTTGTAAAGTTGAAATAAGTAATACTTTCTTGTTTAATCTGTGCAATCAGAAGGT

GTCTTGACCTTCAATTCAATTGGTTTCTTTTAACAAAAATAAACACTGCTAAAAGTTAT**T**

**GTTGTTGCCTCTAACATG**
